# Supplementary material for: Profiling the colonic mucosal response to fecal microbiota transplantation identifies a role for GBP5 in colitis in humans and mice
Source: Nat Commun. 2024 Mar 26;15:2645. doi: 10.1038/s41467-024-46983-5 (PMC10965925; doi:10.1038/s41467-024-46983-5)

Supplementary figure 1

FMT has a substantial effect on colonic mucosal microbiota. A: Alpha diversity indices across groups. Group differences were tested using One-Way ANOVA with Tukey's multiple comparison test. Richness measures were log-transformed prior to testing to fit data into a normal distribution and enable parametric testing. Tx0, baseline (n=26); P8, post-placebo (n=8); Tx8, post-FMT (n=26). Data are presented as mean ± SEM. **B:** Principal coordinate analysis of microbiota beta-diversity. Bray-Curtis similarities were calculated on square-root transformed relative abundances (%) of bacterial operational taxonomic units. Inter-group differences were tested using One-Way ANOSIM, with one-tailed significance computed by permutation.

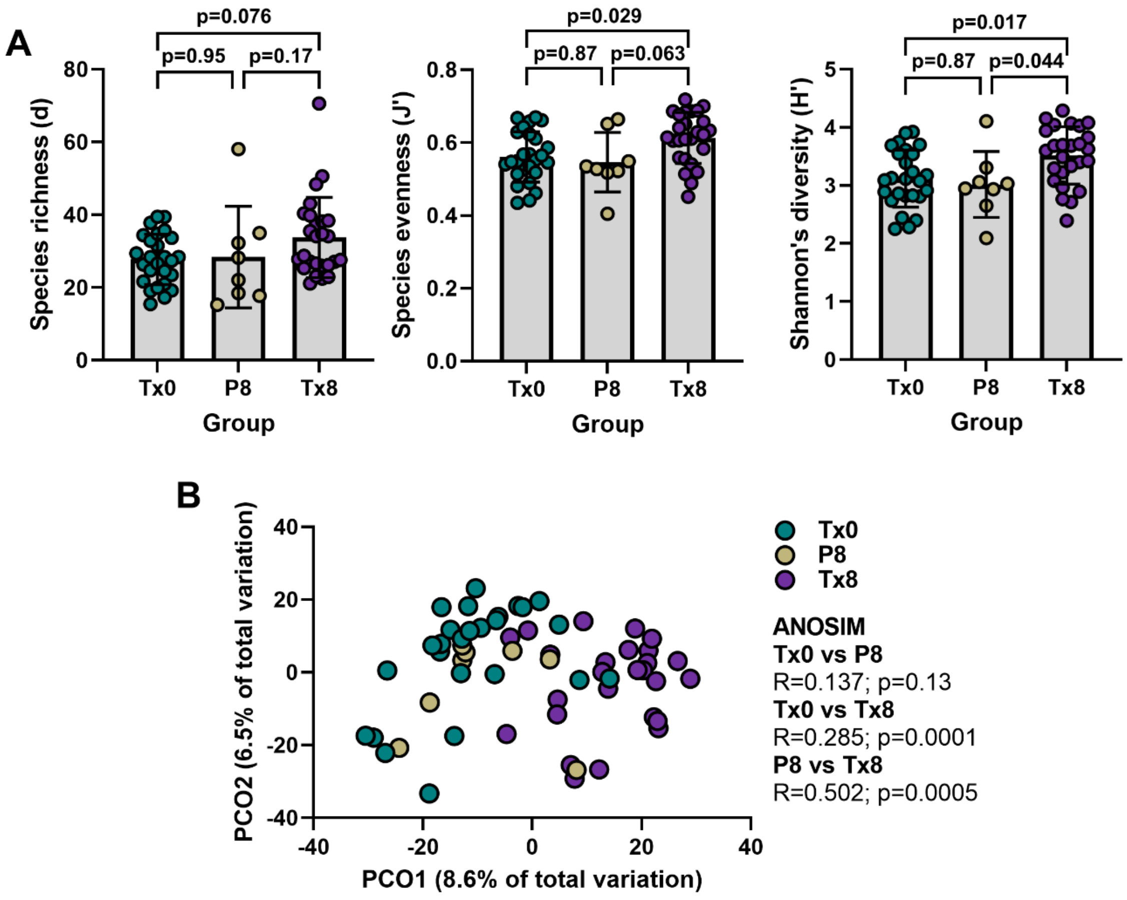

Supplementary figure 2

Quality of transcriptome data. A: SeqMONK read assignment according to genomic location (n=60 samples). **B:** Number of expressed genes per group. Tx0, baseline (n=26); P8, post-placebo (n=8); Tx8, post-FMT (n=26). No differences were found among the groups. **C:** Principal coordinate analysis of Bray-Curtis similarities of inferred cell-types (CIBERSORTx). Differences among groups were tested using One-Way ANOSIM, with one-tailed significance computed by permutation. Tx0N, non-responder baseline (n=13); Tx0Y, responder baseline (n=13); Tx8N, non-responder post-FMT (n=13); Tx8Y, responder post-FMT (n=13); P8, post-placebo (n=8). **D:** Differences in specific cell types as tested using Two-Way repeated measures ANOVA. Cell-types with uncorrected borderline (p<0.07) or significant results were presented as mean ± SEM.

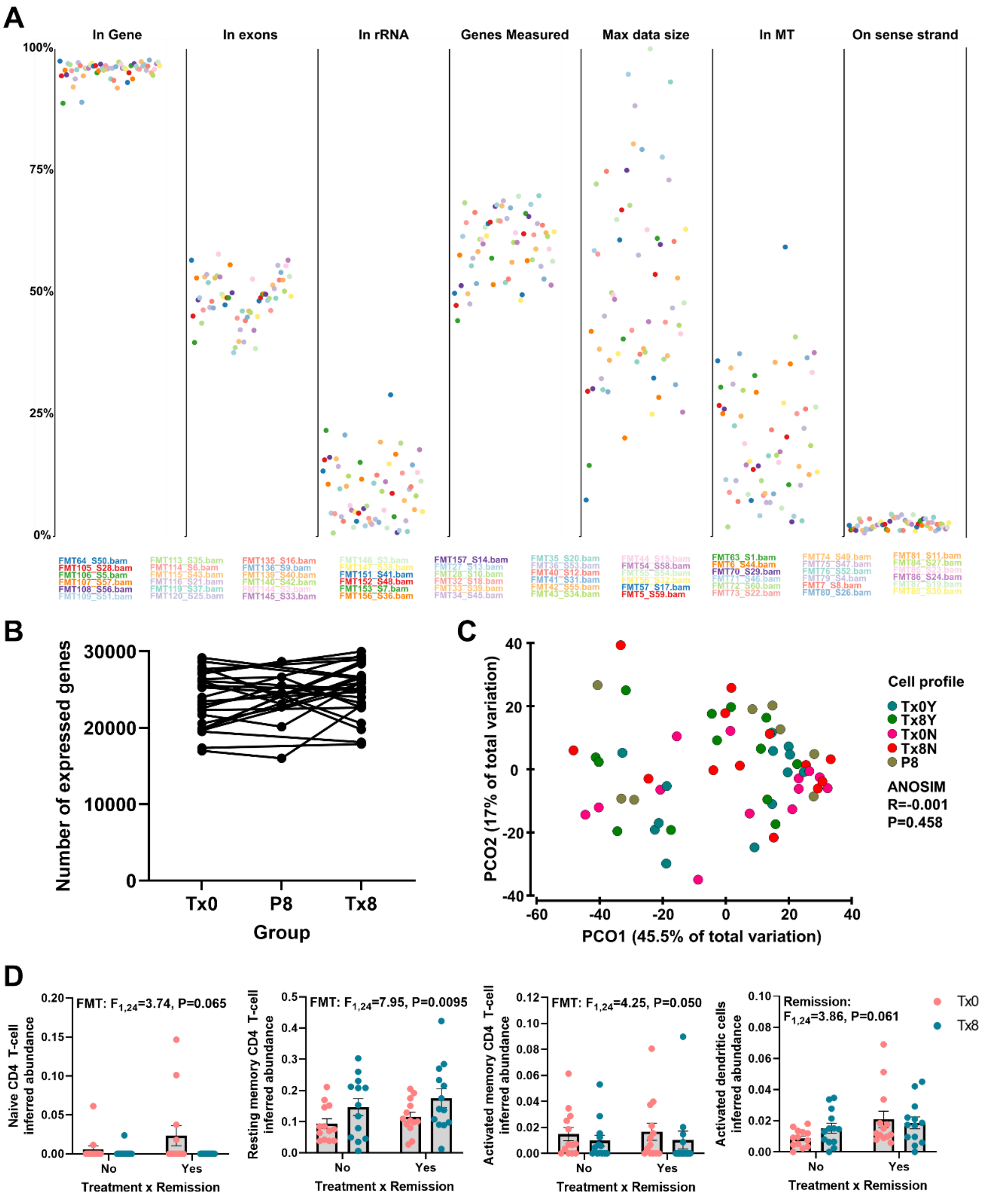

Supplementary figure 3

Differences in colonic mucosal microbiota of responders to FMT when compared to non-responders. A: Species richness and evenness, and **B:** Shannon's diversity index across groups. Group differences were tested using One-Way ANOVA with Holm-Šídák's multiple comparisons test. Richness measures were log-transformed prior to testing to fit data into a normal distribution and enable parametric testing. Tx0N, non-responder baseline (n=13); Tx0Y, responder baseline (n=13); Tx8N, non-responder post-FMT (n=13); Tx8Y, responder post-FMT (n=13). Data are presented as mean ± SEM. **C:** Principal coordinate analysis of microbiota beta-diversity. Bray-Curtis similarities were calculated on square-root transformed relative abundances (%) of bacterial operational taxonomic units. Inter-group differences were tested using Two-Way ANOSIM (week x response), with one-tailed significance computed by permutation.

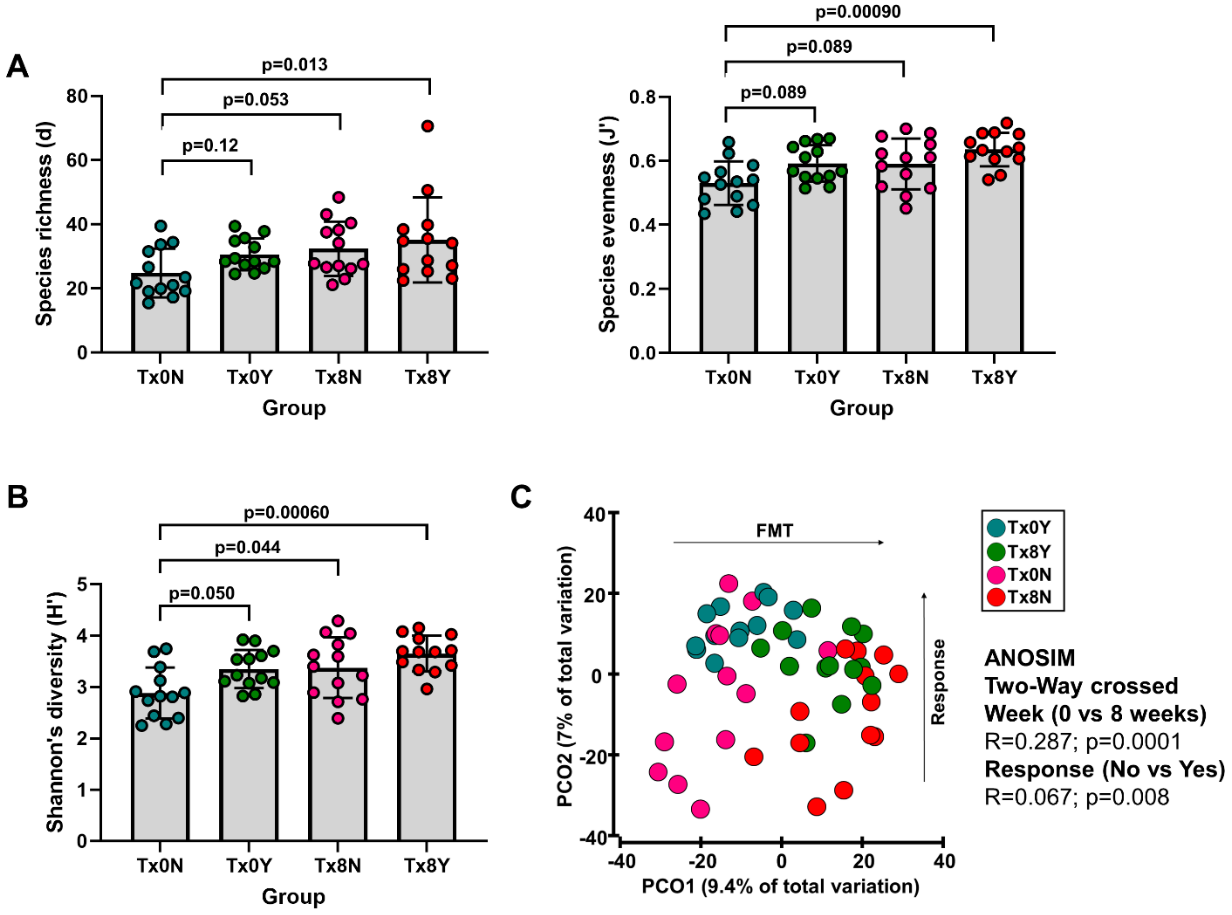

Supplementary figure 4

Both responders and non-responders to FMT have effects on alternative transcript splicing in their colonic mucosa. A: Volcano plot of alternative splicing (delta PSI) in responders following treatment with FMT for 8 weeks. Tx0Y, responder baseline (n=13); Tx8Y, responder post-FMT (n=13). Red, increased differential splicing; Green, decreased differential splicing. **B:** Volcano plot of alternative splicing in non-responders following treatment with FMT for 8 weeks. Tx0N, non-responder baseline (n=13); Tx8N, non-responder post-FMT (n=13). **C:** Significantly regulated KEGG pathways (q<0.05) from list of genes containing alternatively spliced transcripts of responders to FMT. **D:** Alternative splicing event within spleen associated tyrosine kinase SYK leading to inferred differences in protein translation.

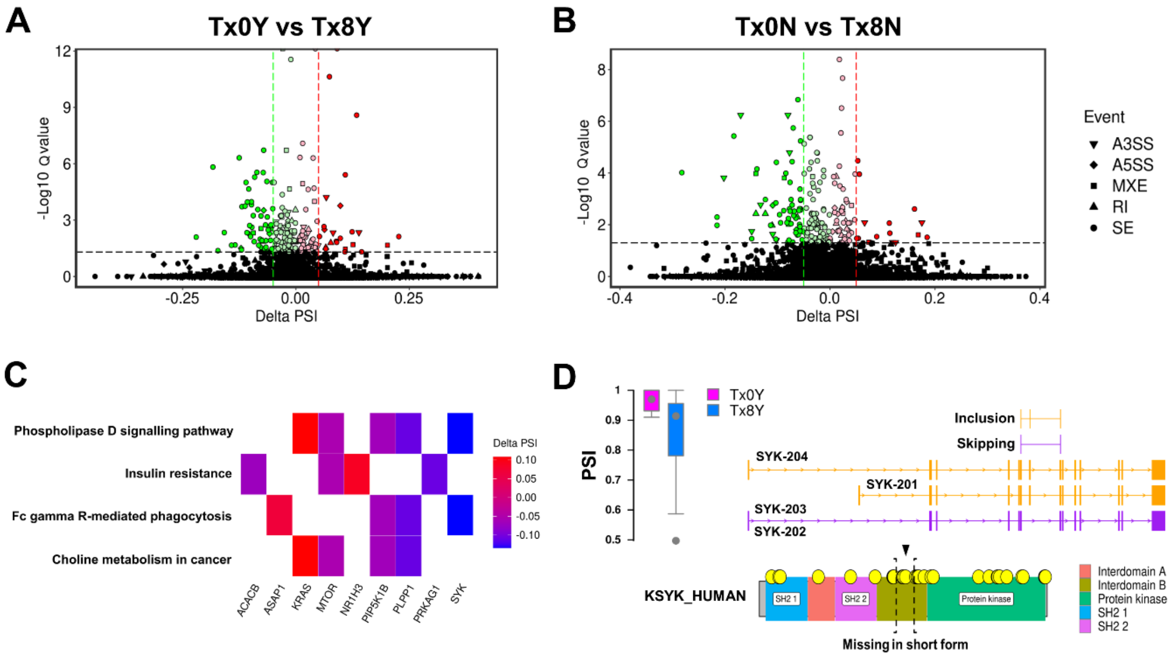

Supplementary figure 5

Correlation between host mucosal markers of response GBP5 and IRF4 with bacterial taxa. The top 100 bacterial OTUs were analyzed against the normalised counts of *GBP5* and *IRF4* and p-values were corrected for false discovery rate. **A:** Pearson correlation between *GBP5* and *Sutterella* OTU29. **B:** Pearson correlation between *IRF4* and *Sutterella* OTU29. P-values were corrected for false discovery rate (q-value) using the Benjamini-Hochberg method.

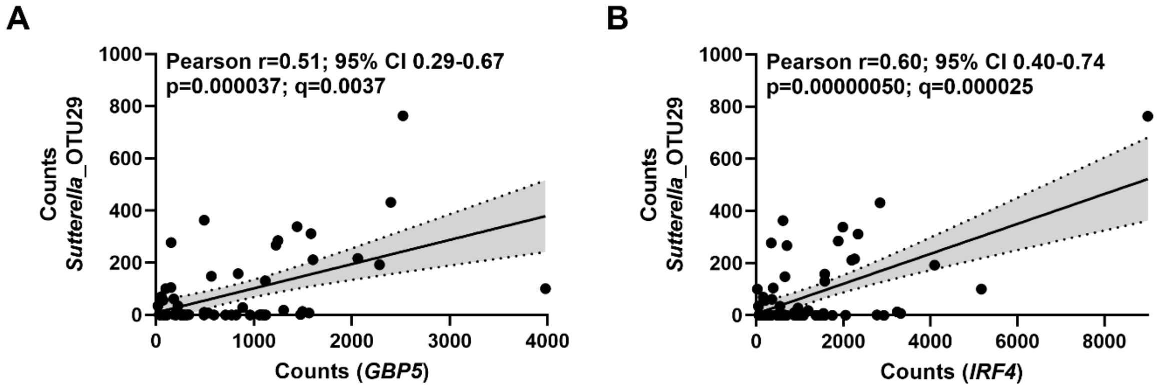

Supplementary figure 6

Differences in fecal microbiota of wild-type (WT) and *Gbp5*$^{-/-}$ littermates. A: Bacterial Shannon's diversity index across groups. No significant differences were observed using One-Way ANOVA with Tukey's multiple comparisons test. Data are presented as mean ± SEM. WT, n=12; *Gbp5*$^{-/-}$ n=14. **B:** Principal coordinate analysis of microbiota beta-diversity. Bray-Curtis similarities were calculated on square-root transformed relative abundances (%) of bacterial OTUs. Inter-group differences were tested using Two-Way ANOSIM (genotype x timepoint), with one-tailed significance computed by permutation. WT, n=12; *Gbp5*$^{-/-}$ n=14. **C:** Shannon's diversity index of mycobiome across groups. No significant differences were observed using Kruskal-Wallis with Dunn's multiple comparisons test. Data are presented as mean ± SEM. WT, n=12; *Gbp5*$^{-/-}$ n=14. **D:** Principal coordinate analysis of mycobiome beta-diversity. Bray-Curtis similarities were calculated on square-root transformed relative abundances (%) of fungal OTUs. Inter-group differences were tested using Two-Way ANOSIM (genotype x timepoint), with one-tailed significance computed by permutation. WT, n=12; *Gbp5*$^{-/-}$ n=14.

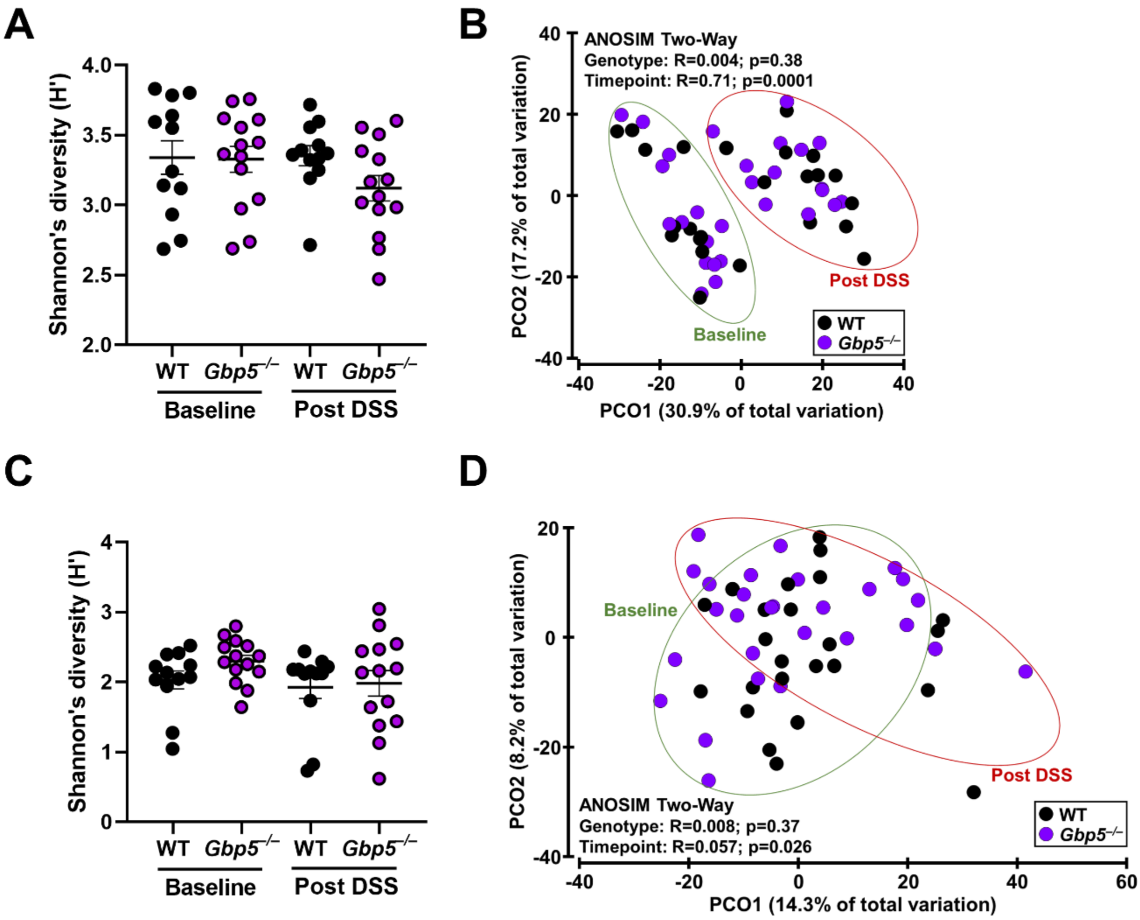

Supplementary figure 7

Differential protein regulation in *Gbp5*$^{-/-}$ and WT littermates. A: Differences in expression and phosphorylation of proteins of interest in littermate WT (n=5) and *Gbp5*$^{-/-}$ (n=5) mice at day 10. Lane volume was employed for densitometry and differences were tested using two-tailed unpaired t-tests. NS, not statistically significant (all p>0.17); **p<0.01 (p=0.0099). Data are presented as mean ± SEM. **B:** Immunohistochemical staining of claudin 2, β-Actin and DAPI in the colon tissue of untreated WT mice (day 0; n=3) or DSS-treated WT mice (day 10; n=3) and quantification of claudin 2-positive tissue area over total colon tissue area. Scale bar 50 µm. Each symbol represents an individual mouse. NS, not statistically significant (p=0.065); *p<0.05 by two-way ANOVA with Šídák's multiple comparisons test (middle: p=0.0375; distal: p=0.0158). Data are presented as mean ± SEM.

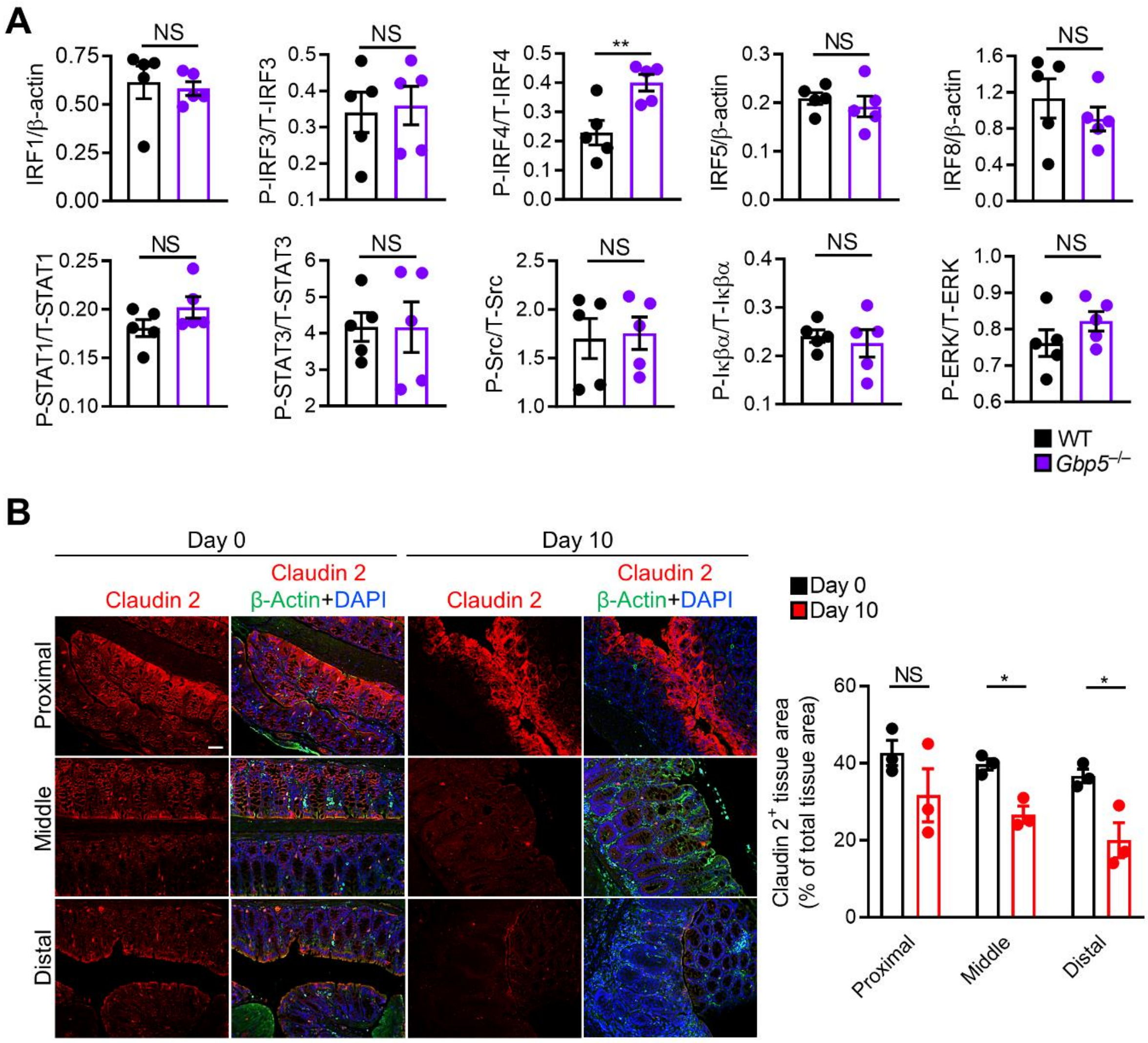

Supplementary figure 8

Description of study cohort. A: 85 patients were recruited of which 81 were treated. 21 patients achieved the primary outcome (n=11 blinded; n=10 open-label). 3/21 responders were recruited at site 3 where no research samples were collected and 1/21 was excluded as they had initially achieved the primary outcome on placebo, which was deemed a confound given that this patient started FMT in remission. RNA from n=17 responders were quality checked and n=4 patients had at least 1 sample that was of insufficient quality. The remaining n=13 responders were matched 1:1 with n=13 non-responders according to baseline disease activity which was considered the most influential confounder to the transcriptome (total n=26; 12 female). All post-placebo (P8) samples from the n=26 patients (n=8) were included. **B:** Total Mayo and Ulcerative Colitis Endoscopic Index of Severity (UCEIS) scores at baseline (Tx0) for selected patients. Differences were assessed using two-tailed unpaired t-tests. Data are presented as mean ± SEM.

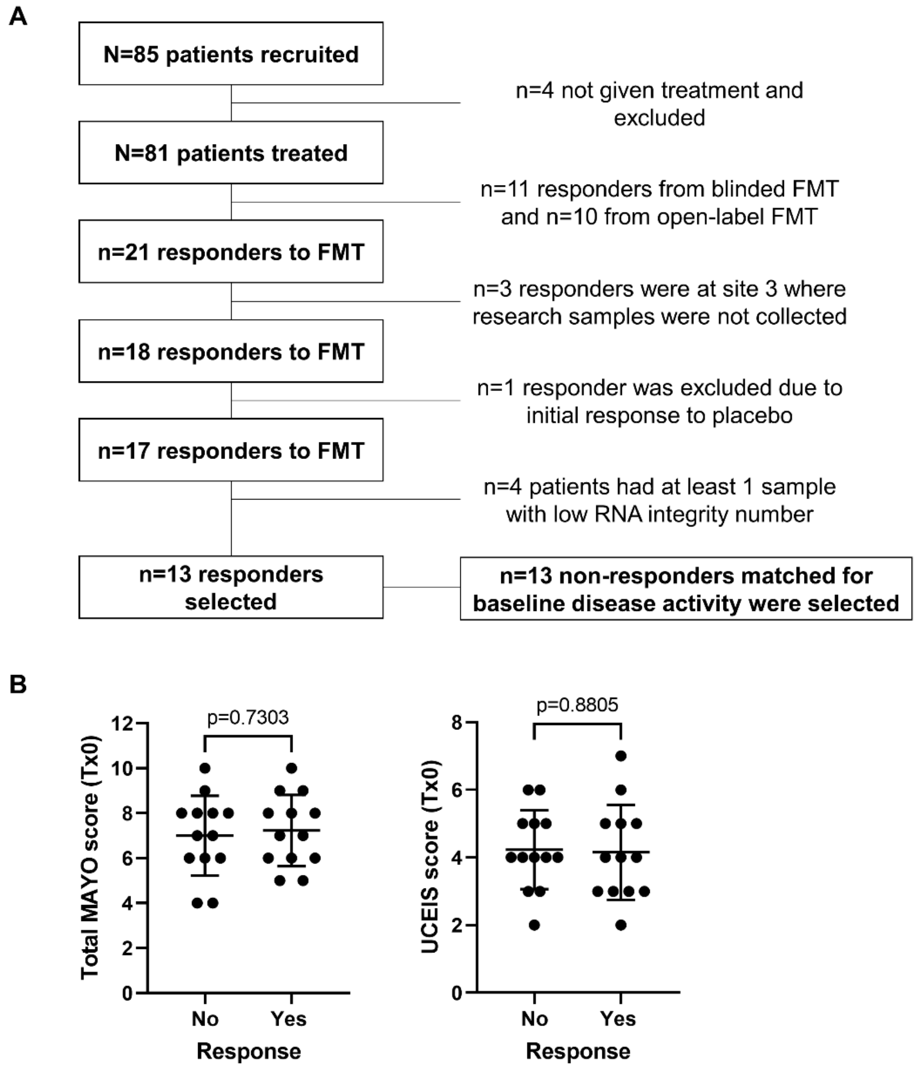

Supplement: Supplementary file 1 — Supplementary Information [file 41467_2024_46983_MOESM1_ESM.pdf]
