# Supplementary material for: Profiling the colonic mucosal response to fecal microbiota transplantation identifies a role for GBP5 in colitis in humans and mice
Source: Nat Commun. 2024 Mar 26;15:2645. doi: 10.1038/s41467-024-46983-5 (PMC10965925; doi:10.1038/s41467-024-46983-5)
Supplement: Supplementary file 3 — Description of Additional Supplementary Files [file 41467_2024_46983_MOESM3_ESM.pdf]

Description of Additional Supplementary Files

Title: Supplementary Data 1

Description: Total number of reads per sample.

Title: Supplementary Data 2

Description: Differentially expressed genes following FMT (Tx8 vs Tx0; Q<0.1) as identified by DESeq2. P-values were corrected for false discovery rate (q-value) using the Benjamini-Hochberg method.

Title: Supplementary Data 3

Description: Differentially expressed genes following placebo (P8 vs Tx0; Q<0.1) as identified by DESeq2. P-values were corrected for false discovery rate (q-value) using the Benjamini-Hochberg method.

Title: Supplementary Data 4

Description: Differentially expressed genes in responders following FMT (Tx8Y vs Tx0Y; Q<0.1) using an unpaired analysis. Transcripts not concordant with the paired analysis (Q<0.1) were highlighted in red. Genes were identified by DESeq2 and p-values were corrected for false discovery rate (q-value) using the Benjamini-Hochberg method.

Title: Supplementary Data 5

Description: Differentially expressed genes in responders following FMT (Tx8Y vs Tx0Y; Q<0.1) using a paired analysis. Genes were identified by DESeq2 and p-values were corrected for false discovery rate (q-value) using the Benjamini-Hochberg method.

Title: Supplementary Data 6

Description: Differentially expressed genes in non-responders following FMT (Tx8N vs Tx0N; Q<0.1) using a paired analysis. Genes were identified by DESeq2 and p-values were corrected for false discovery rate (q-value) using the Benjamini-Hochberg method.

Title: Supplementary Data 7

Description: Summary of splicing events within the data.

Title: Supplementary Data 8

Description: Differentially spliced events in responders following FMT (Tx8Y vs Tx0Y; Q<0.05).

Title: Supplementary Data 9

Description: Differentially spliced events in non-responders following FMT (Tx8N vs Tx0N; Q<0.05).
