# Supplementary material for: Profiling the colonic mucosal response to fecal microbiota transplantation identifies a role for GBP5 in colitis in humans and mice
Source: Nat Commun. 2024 Mar 26;15:2645. doi: 10.1038/s41467-024-46983-5 (PMC10965925; doi:10.1038/s41467-024-46983-5)
Supplement: Supplementary file 5 — Reporting Summary [file 41467_2024_46983_MOESM5_ESM.pdf]

nature portfolio

Corresponding author(s): Nadeem Kaakoush

Last updated by author(s): Feb 26, 2024

Reporting Summary

Nature Portfolio wishes to improve the reproducibility of the work that we publish. This form provides structure for consistency and transparency in reporting. For further information on Nature Portfolio policies, see our Editorial Policies and the Editorial Policy Checklist.

Statistics

For all statistical analyses, confirm that the following items are present in the figure legend, table legend, main text, or Methods section.

n/a	Confirmed	
☐	☒	The exact sample size (*n*) for each experimental group/condition, given as a discrete number and unit of measurement
☐	☒	A statement on whether measurements were taken from distinct samples or whether the same sample was measured repeatedly
☐	☒	The statistical test(s) used AND whether they are one- or two-sided<br>*Only common tests should be described solely by name; describe more complex techniques in the Methods section.*
☒	☐	A description of all covariates tested
☐	☒	A description of any assumptions or corrections, such as tests of normality and adjustment for multiple comparisons
☐	☒	A full description of the statistical parameters including central tendency (e.g. means) or other basic estimates (e.g. regression coefficient) AND variation (e.g. standard deviation) or associated estimates of uncertainty (e.g. confidence intervals)
☐	☒	For null hypothesis testing, the test statistic (e.g. *F*, *t*, *r*) with confidence intervals, effect sizes, degrees of freedom and *P* value noted<br>*Give P values as exact values whenever suitable.*
☒	☐	For Bayesian analysis, information on the choice of priors and Markov chain Monte Carlo settings
☒	☐	For hierarchical and complex designs, identification of the appropriate level for tests and full reporting of outcomes
☐	☒	Estimates of effect sizes (e.g. Cohen's *d*, Pearson's *r*), indicating how they were calculated

Our web collection on statistics for biologists contains articles on many of the points above.

Software and code

Policy information about availability of computer code

Data collection	Illumina NovaSeq 6000<br>Illumina MiSeq<br>BaseSpace cloud platform<br>ChemiDoc™ Imaging System (Bio-Rad)<br>Zeiss Axio Observer microscope
Data analysis	fastqc v0.11.5<br>Salmon v1.2.1<br>SeqMonk v1.47.1<br>CIBERSORTx<br>Primer-e v6<br>DESeq2 v1.30.1<br>GAGE v2.40.2<br>rMATs v4.0.2<br>python v2.7.15<br>gsl v1.16<br>star v2.7.2b<br>samtools v1.10<br>Enrichr

ggplot2 v3.3.3
maser v1.8.0
drawProteins v1.10.0
GraphPad Prism v9
Image Lab software (Bio-Rad)
Fiji (National Institutes of Health, USA)
Mothur v1.44.2
vsearch v2.13.3

For manuscripts utilizing custom algorithms or software that are central to the research but not yet described in published literature, software must be made available to editors and reviewers. We strongly encourage code deposition in a community repository (e.g. GitHub). See the Nature Portfolio guidelines for submitting code & software for further information.

Data

Policy information about availability of data

All manuscripts must include a data availability statement. This statement should provide the following information, where applicable:

- Accession codes, unique identifiers, or web links for publicly available datasets
- A description of any restrictions on data availability
- For clinical datasets or third party data, please ensure that the statement adheres to our policy

The reused human mucosal microbiota data have been deposited in the European Nucleotide Archive (ENA) database under accession code PRJEB26473 [https://www.ebi.ac.uk/ena/browser/view/PRJEB26473]. The mouse fecal microbiota data (both 16S rRNA gene and ITS2 region) generated in this study have been deposited in ENA under accession codes PRJEB55997 [https://www.ebi.ac.uk/ena/browser/view/PRJEB55997] for 16S rRNA gene and PRJEB72841 [https://www.ebi.ac.uk/ena/browser/view/PRJEB72841] for the ITS2 region. They can also be accessed in the Zenodo database under accession code 10128069 [https://zenodo.org/records/10128069]. The RNA sequencing data generated in this study have been deposited in ENA under accession code PRJEB73377 [https://www.ebi.ac.uk/ena/browser/view/PRJEB73377]. The processed RNA sequencing data generated in this study have been deposited in the Zenodo database under accession code 10622773 [https://zenodo.org/records/10622773]. Databases used in this study can be accessed through the following links: the human GRCh38 reference genome [https://www.ncbi.nlm.nih.gov/datasets/genome/GCF_000001405.26/], RDP v18 [https://mothur.org/wiki/rdp_reference_files/], SILVA [https://mothur.org/wiki/silva_reference_files/], and UNITE v6 [https://mothur.org/wiki/unite_its_database/]. All other data are provided in the main text, supplementary information, or source data file.

Research involving human participants, their data, or biological material

Policy information about studies with human participants or human data. See also policy information about sex, gender (identity/presentation), and sexual orientation and race, ethnicity and racism.

Reporting on sex and gender	Subjects from both male and female sex were recruited to the randomized clinical trial. On final selection of samples that were analyzed in this study, there were 12 female and 14 male subjects. The transcriptome data was not analyzed according to sex as this is a double-blind, randomized, clinical trial and it is not powered to detect sex differences. There are no studies on gender reported in this work.
Reporting on race, ethnicity, or other socially relevant groupings	We did not account for race, ethnicity, or other socially relevant groupings in our analyses.
Population characteristics	We conducted a multicentre, double-blind, randomized, placebo-controlled trial at three hospitals in Australia. We randomly allocated patients with active ulcerative colitis (Mayo score 4-10) in a 1:1 ratio, using a pre-established randomization list, to either fecal microbiota transplantation or placebo colonoscopic infusion, followed by enemas 5 days per week for 8 weeks. The primary outcome was steroid-free clinical remission with endoscopic remission or response (Mayo score ≤2, all subscores ≤1, and ≥1 point reduction in endoscopy subscore) at week 8. The study protocol is published in Paramsothy, S., et al. Multidonor intensive faecal microbiota transplantation for active ulcerative colitis: a randomised placebo-controlled trial. Lancet 389, 1218-1228 (2017).<br><br>From November, 2013, to May, 2015, 85 patients were enrolled to our trial, of whom 42 were randomly assigned faecal microbiota transplantation and 43 were allocated placebo. One patient assigned faecal microbiota transplantation and three allocated placebo did not receive study treatment and were excluded from the analysis.<br><br>Of the 85 patients enrolled, n=26 were selected for RNA sequencing (please see Recruitment below). Of these 26 patients (n=12 female) selected for analysis, mean age ± SEM=40.8 ± 2.3 years (95% CI=36.0-45.6). n=18 (9 female) and n=8 (3 female) patients were in the blinded (directly on FMT) and open label (i.e., initially on placebo) arms, respectively.
Recruitment	85 subjects were recruited to the trial of which 81 were treated. 21 subjects achieved the primary outcome (n=11 blinded; n=10 open-label). 3/21 responders were recruited at site 3 where no research samples were collected and 1/21 was excluded as they had initially achieved the primary outcome on placebo, which was deemed a confound given that this patient started FMT in remission. RNA from n=17 responders were quality checked and n=4 subjects had at least 1 sample that was of insufficient quality. The remaining n=13 responders were matched 1:1 with n=13 non-responders according to baseline disease activity which was considered the most influential confounder to the transcriptome (total n=26; 12 female). All post-placebo (P8) samples from the n=26 subjects (n=8) were included. This totaled to n=60 samples.
Ethics oversight	St Vincent's Hospital Sydney Human Research Ethics Committee

Note that full information on the approval of the study protocol must also be provided in the manuscript.

Field-specific reporting

Please select the one below that is the best fit for your research. If you are not sure, read the appropriate sections before making your selection.

[x] Life sciences [ ] Behavioural & social sciences [ ] Ecological, evolutionary & environmental sciences

For a reference copy of the document with all sections, see nature.com/documents/nr-reporting-summary-flat.pdf

Life sciences study design

All studies must disclose on these points even when the disclosure is negative.

Sample size	No statistical methods were used to predetermine sample sizes. Sufficient quality RNA for shotgun sequencing was available from n=13 responders. These were matched 1:1 with n=13 non-responders according to baseline disease activity as this was considered the most influential confounder to the transcriptome. The total number of patients recruited was n=26 (12 female). All post-placebo (P8) samples from the n=26 subjects (n=8) were included which totaled to n=60 samples. These sample sizes were sufficient given the substantial differences in the clinical characteristics of the groups being compared (active ulcerative colitis vs disease in remission). Specific details of sample size and statistical analysis are presented in the figure legends.
Data exclusions	Samples were excluded from sequencing due to low RNA integrity score. All samples that were sequenced were analyzed and none were excluded from downstream statistical analyses. A detailed explanation of the sample selection is provided in Supplementary figure 8.
Replication	The experimental findings were reliably reproduced. See details in figure legends.
Randomization	We randomly allocated patients with active ulcerative colitis (Mayo score 4-10) in a 1:1 ratio, using a pre-established randomization list, to either fecal microbiota transplantation or placebo colonoscopic infusion. For the current study, we selected 13 responders and matched them 1:1 with 13 non-responders based on their baseline disease activity.<br><br>For the animal experiments, animals were assigned to group based on their genetic background. All samples derived from these animals were assigned to their group based on their genotype and the time they were collected (day 0 vs day 10).
Blinding	The trial was double-blinded. Patients, treating clinicians, and other study staff were unaware of the assigned treatment. For the current study, we attempted to analyze the highest number of responders to FMT in the trial, matched 1:1 with non-responders to FMT. Samples selected for RNA sequencing were selected based on their RNA quality and the patient subgroup they were derived from. Thus, blinding at this stage was not possible.<br><br>All other results presented are based on objective analysis of the capture data, without subjective interpretation. Hence, blinding was not required for these analyses.

Reporting for specific materials, systems and methods

We require information from authors about some types of materials, experimental systems and methods used in many studies. Here, indicate whether each material, system or method listed is relevant to your study. If you are not sure if a list item applies to your research, read the appropriate section before selecting a response.

Materials & experimental systems

n/a	Involved in the study	
[ ]	[x]	Antibodies
[x]	[ ]	Eukaryotic cell lines
[x]	[ ]	Palaeontology and archaeology
[ ]	[x]	Animals and other organisms
[x]	[ ]	Clinical data
[x]	[ ]	Dual use research of concern
[x]	[ ]	Plants

Methods

n/a	Involved in the study	
[x]	[ ]	ChIP-seq
[x]	[ ]	Flow cytometry
[x]	[ ]	MRI-based neuroimaging

Antibodies

Antibodies used	Primary antibodies used for immunoblotting were β-actin (Cell Signalling Technology, #4970), GBP5 (1:1000), Phospho-Src (Tyr527) (New England Biolabs, #2105S), Src (New England Biolabs, #2108S), Phospho-ERK1/2 Thr202/Tyr204 (Cell Signalling Technology, #9101), ERK1/2 (Cell Signalling Technology, #9102), Phospho-IkB (Cell Signaling Technology, #2859), IkB (Cell Signaling Technology, #9242), IRF1 (Cell Signaling Technology, #8478), Phospho-IRF3 (Cell Signaling Technology, #4947), IRF3 (Cell Signaling Technology, #4302), Phospho-IRF4 Tyr122/Tyr125 (ThermoFisher Scientific, #PA5105214), IRF4 (New England Biolabs, #4948S), IRF5 (Abcam, #ab33478), IRF8 (Cell Signaling Technology, #5628), Phospho-STAT1 (Cell Signaling Technology, #9167), STAT1 (Cell Signaling Technology, #14994), Phospho-STAT3 (Cell Signaling Technology, #9145), and STAT3 (Cell Signaling Technology, #9139). Secondary antibodies used for immunoblotting were Peroxidase AffiniPure (Jackson ImmunoResearch, #111-035-045) and Peroxidase AffiniPure

	(Jackson ImmunoResearch, #115-035-146).<br><br>Primary antibodies employed for immunohistochemistry targeted claudin 2 (1:100; Abcam, ab53032) and β-actin (1:200; Abcam, ab8226). Secondary antibodies used were Rhodamine RedTM-X conjugate (1:300; Jackson ImmunoResearch, 111-295-144) and Alexa Fluor 488 AffiniPure (1:300; Jackson ImmunoResearch, 115-545-003).
Validation	The primary antibody targeting GBP5 was developed by Degrandi, D., et al. [J Immunol 179, 7729-7740 (2007)] and has been used elsewhere [PMID: 35906252].<br><br>All other antibodies used in this study were commercially validated. Validations are available on the manufacturer's website:<br>β-actin (https://www.cellsignal.com/products/primary-antibodies/b-actin-13e5-rabbit-mab/4970)<br>Phospho-Src (https://www.cellsignal.com/product/productDetail.jsp?productId=2105)<br>Src (https://www.cellsignal.com/product/productDetail.jsp?productId=2108)<br>Phospho-ERK1/2 (https://www.cellsignal.com/products/primary-antibodies/phospho-p44-42-mapk-erk1-2-thr202-tyr204-antibody/9101)<br>ERK1/2 (https://www.cellsignal.com/products/primary-antibodies/p44-42-mapk-erk1-2-antibody/9102)<br>Phospho-IkB (https://www.cellsignal.com/products/primary-antibodies/phospho-ikba-ser32-14d4-rabbit-mab/2859)<br>IkB (https://www.cellsignal.com/products/primary-antibodies/ikba-antibody/9242)<br>IRF1 (https://www.cellsignal.com/products/primary-antibodies/irf-1-d5e4-xp-rabbit-mab/8478)<br>Phospho-IRF3 (https://www.cellsignal.com/products/primary-antibodies/phospho-irf-3-ser396-4d4g-rabbit-mab/4947)<br>IRF3 (https://www.cellsignal.com/products/primary-antibodies/stat3-124h6-mouse-mab/4302)<br>Phospho-IRF4 (https://www.thermofisher.com/antibody/product/Phospho-IRF4-Tyr122-Tyr125-Antibody-Polyclonal/PA5-105214)<br>IRF4 (https://www.cellsignal.com/product/productDetail.jsp?productId=4948)<br>IRF5 (https://www.abcam.com/en-au/products/primary-antibodies/irf5-antibody-10t1-ab33478)<br>IRF8 (https://www.cellsignal.com/products/primary-antibodies/stat3-124h6-mouse-mab/5628)<br>Phospho-STAT1 (https://www.cellsignal.com/products/primary-antibodies/stat3-124h6-mouse-mab/9167)<br>STAT1 (https://www.cellsignal.com/products/primary-antibodies/stat3-124h6-mouse-mab/14994)<br>Phospho-STAT3 (https://www.cellsignal.com/products/primary-antibodies/stat3-124h6-mouse-mab/9145)<br>STAT3 (https://www.cellsignal.com/products/primary-antibodies/stat3-124h6-mouse-mab/9139)<br>Peroxidase AffiniPure Goat Anti-Rabbit IgG (H+L) (https://www.jacksonimmuno.com/catalog/products/111-035-045)<br>Peroxidase AffiniPure Goat Anti-Mouse IgG (H+L) (https://www.jacksonimmuno.com/catalog/products/115-035-146)<br>claudin 2 (https://www.abcam.com/en-au/products/primary-antibodies/claudin-2-antibody-ab53032)<br>β-actin (https://www.abcam.com/en-au/products/primary-antibodies/beta-actin-antibody-mabcam-8226-loading-control-ab8226)<br>Rhodamine RedTM-X conjugate (https://www.jacksonimmuno.com/catalog/products/111-295-144)<br>Alexa Fluor 488 AffiniPure (https://www.jacksonimmuno.com/catalog/products/115-545-003)

Animals and other research organisms

Policy information about studies involving animals; ARRIVE guidelines recommended for reporting animal research, and Sex and Gender in Research

Laboratory animals	Mice with a genomic deletion of GBP5 (called Gbp5–/–) were generated by Cas9/CRISPR-mediated genome editing technology and characterized as previously described (Feng, S., et al. Pathogen-selective killing by guanylate-binding proteins as a molecular mechanism leading to inflammasome signaling. Nat Commun 13, 4395 (2022)). All mice are on the C57BL/6NcrlAnu background. Mice were bred and maintained at The Australian National University under specific pathogen-free conditions.<br><br>Mouse cages receive 16 fresh HEPA-filtered air changes per hour within rooms kept at 18-22 degree Celsius and 30% humidity. A 12 hour light-dark cycle is automatically controlled in each room. Female mice between 8-10 weeks old were used for experiments.
Wild animals	No wild animals were used in this study.
Reporting on sex	Female littermate wild-type (WT; n=12) and Gbp5–/– mice (n=14) of 8-10 weeks were given 1.5% dextran sulfate sodium (DSS) (MP Biomedicals, #160110) in their drinking water for 6 days, followed by regular drinking water for 4 days. Mice were ethically culled on day 10. Sex based differences in susceptibility to DSS-induced colitis have been previously reported in mice (Bábíčková, J., et al. Sex Differences in Experimentally Induced Colitis in Mice: a Role for Estrogens. Inflammation 38, 1996-2006 (2015)); thus, we focused our studies on one sex, with the litter-mate WT controls being sex-matched with the Gbp5-/- mice.
Field-collected samples	No field-collected samples were used in this study.
Ethics oversight	All animal studies were conducted in accordance with the Protocol Number A2020/18 approved by The Australian National University Animal Experimentation Ethics Committee.

Note that full information on the approval of the study protocol must also be provided in the manuscript.

Plants

Seed stocks	N/A
Novel plant genotypes	N/A
Authentication	N/A
