# Supplementary material for: Profiling the colonic mucosal response to fecal microbiota transplantation identifies a role for GBP5 in colitis in humans and mice
Source: Nat Commun. 2024 Mar 26;15:2645. doi: 10.1038/s41467-024-46983-5 (PMC10965925; doi:10.1038/s41467-024-46983-5)
Supplement: Supplementary file 6 — Source Data [file 41467_2024_46983_MOESM6_ESM.zip › Source data/Uncropped Blots_Nat Comm.pptx]

## Slide 1
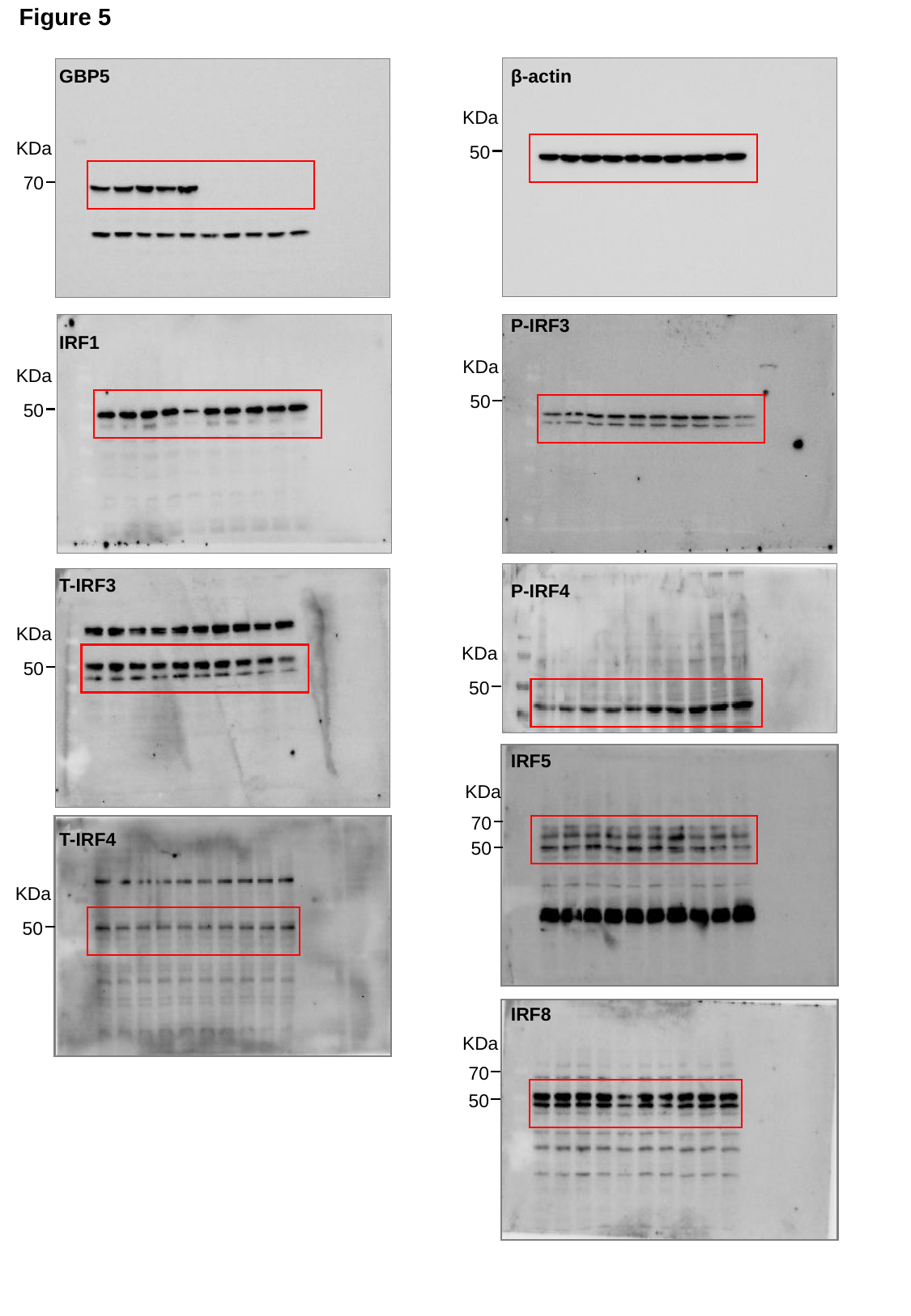

Figure 5
GBP5
β-actin
KDa
KDa
50
70
P-IRF3
IRF1
KDa
KDa
50
50
T-IRF3
P-IRF4
KDa
50
KDa
50
IRF5
KDa
70
T-IRF4
50
KDa
50
IRF8
KDa
70
50

## Slide 2
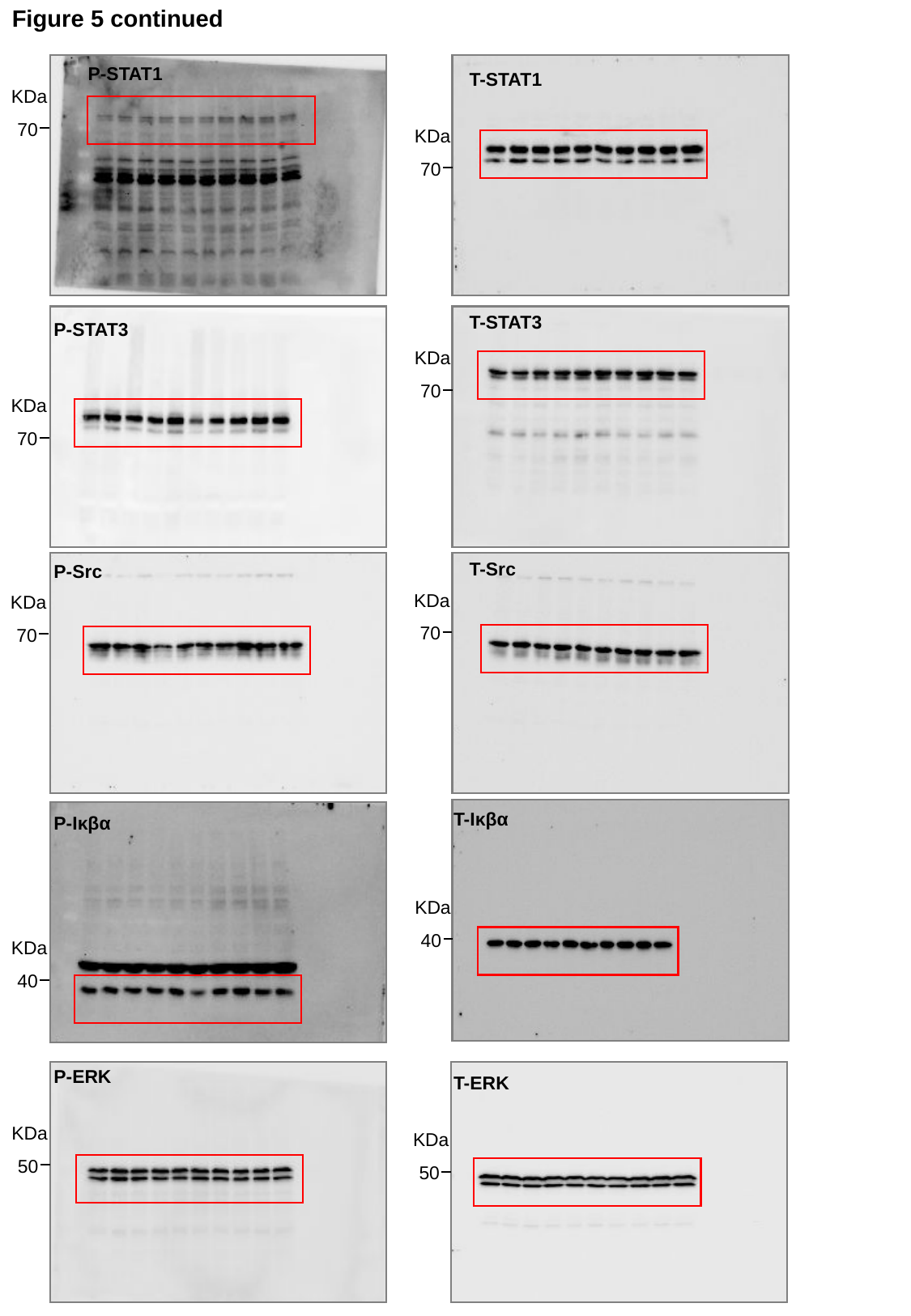

Figure 5 continued
P-STAT1
T-STAT1
KDa
70
KDa
70
T-STAT3
P-STAT3
KDa
70
KDa
70
T-Src
P-Src
KDa
KDa
70
70
T-Iκβα
P-Iκβα
KDa
40
KDa
40
P-ERK
T-ERK
KDa
KDa
50
50
