# Supplementary figures and images for: Profiling the colonic mucosal response to fecal microbiota transplantation identifies a role for GBP5 in colitis in humans and mice
Source: Nat Commun. 2024 Mar 26;15:2645. doi: 10.1038/s41467-024-46983-5 (PMC10965925; doi:10.1038/s41467-024-46983-5)

**Figure 5**

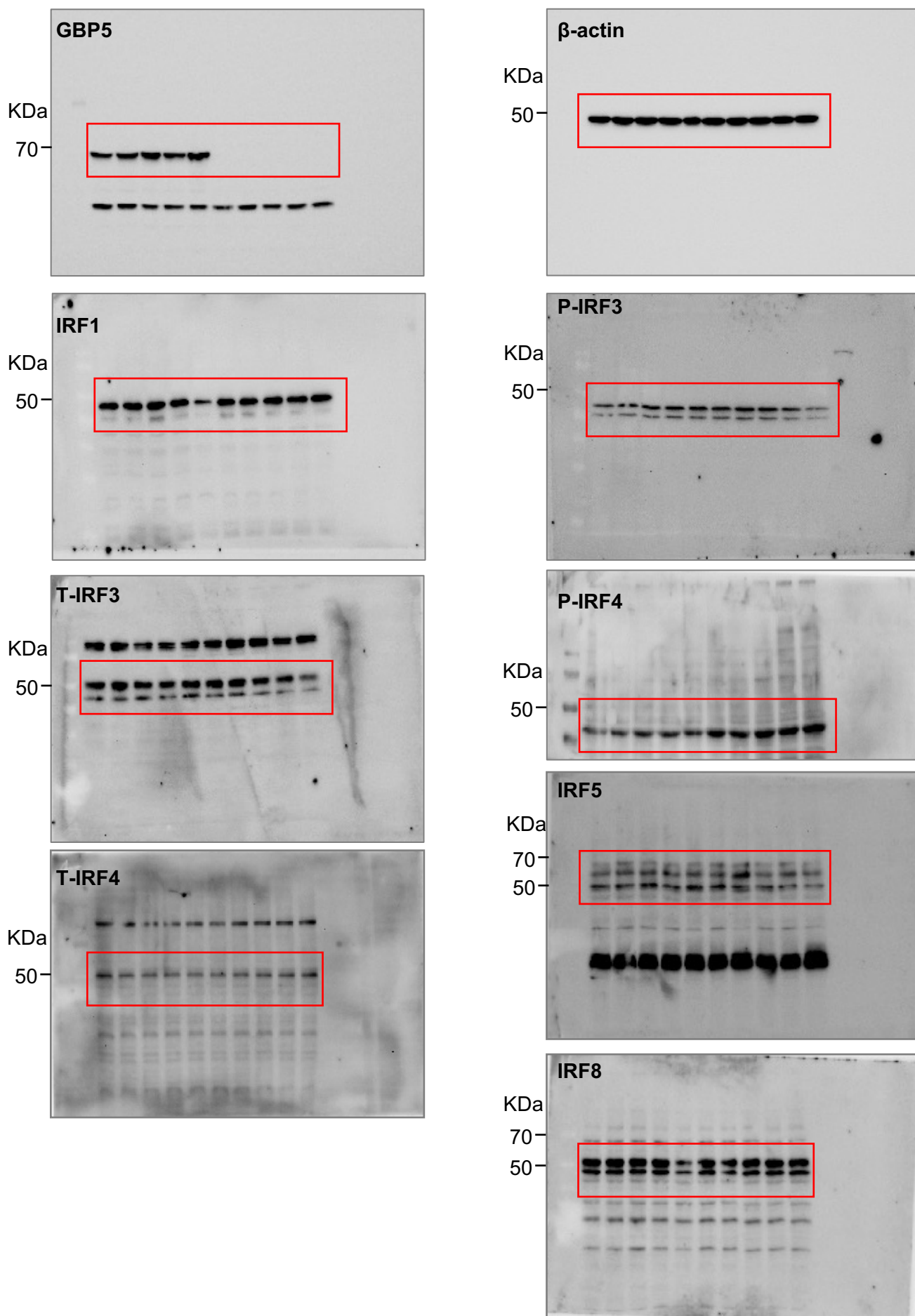

**Figure 5 continued**

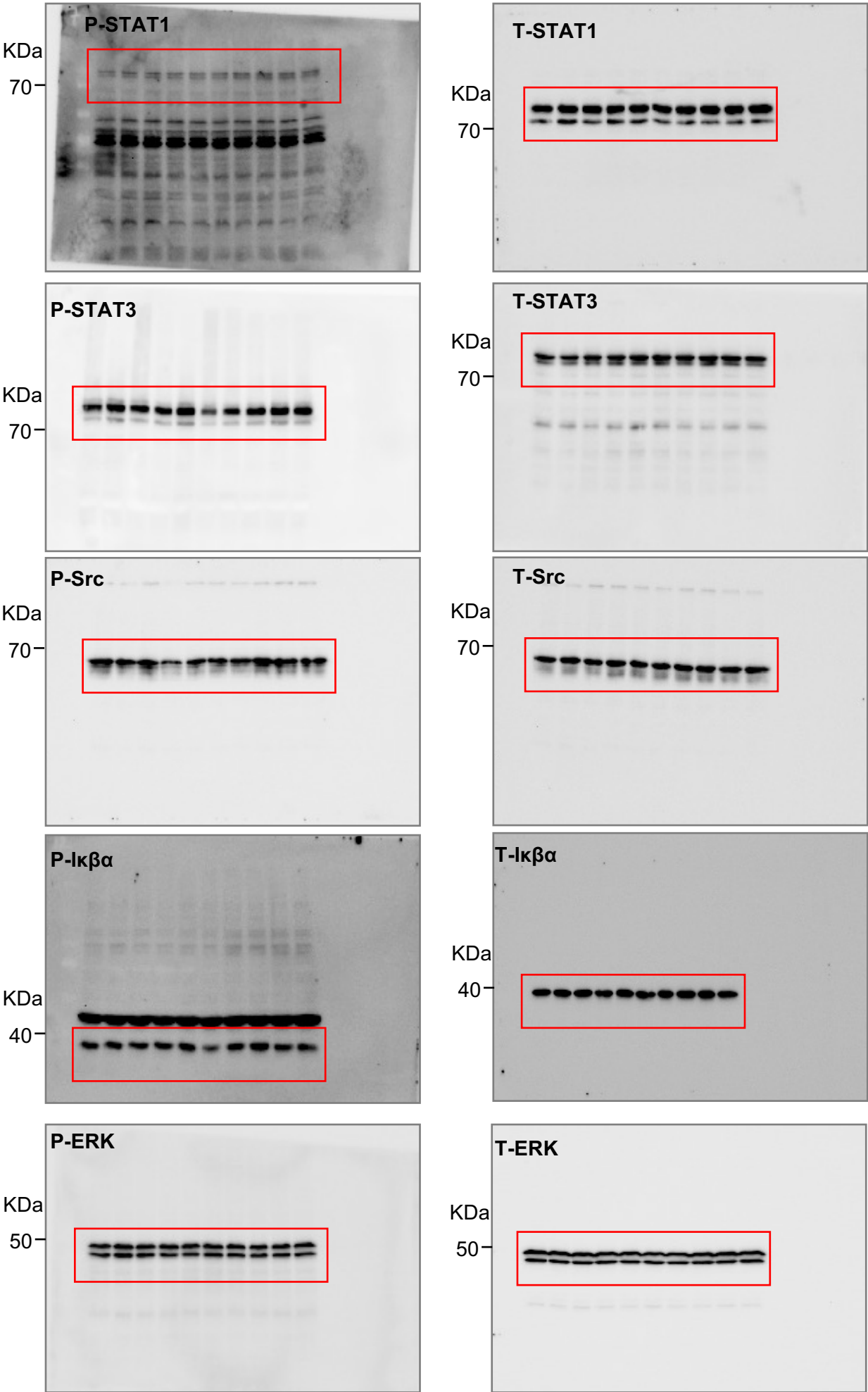

Supplement: Supplementary file 6 — Source Data [file 41467_2024_46983_MOESM6_ESM.zip › Source data/Uncropped Blots_Nat Comm.pdf]
